# Supplementary figures and images for: Prognostic value of KRAS mutation status in colorectal cancer patients: a population-based competing risk analysis
Source: PeerJ. 2020 Jun 1;8:e9149. doi: 10.7717/peerj.9149 (PMC7271887; doi:10.7717/peerj.9149)

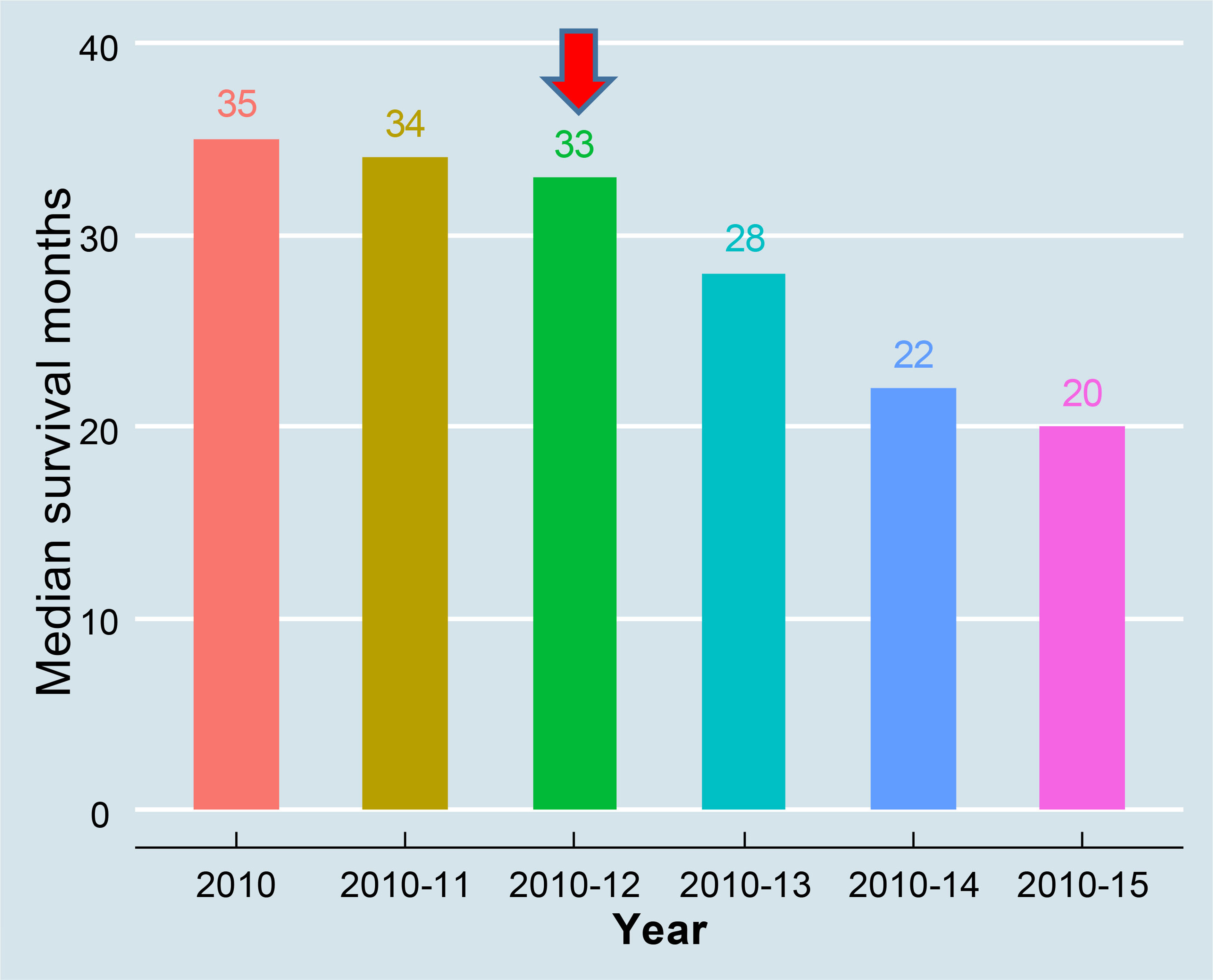

Supplement: Supplemental Information 1 — The current study included with 2010–2012 group (arrow), which exhibited similar median survival time as 2010 group. [file peerj-08-9149-s001.png]

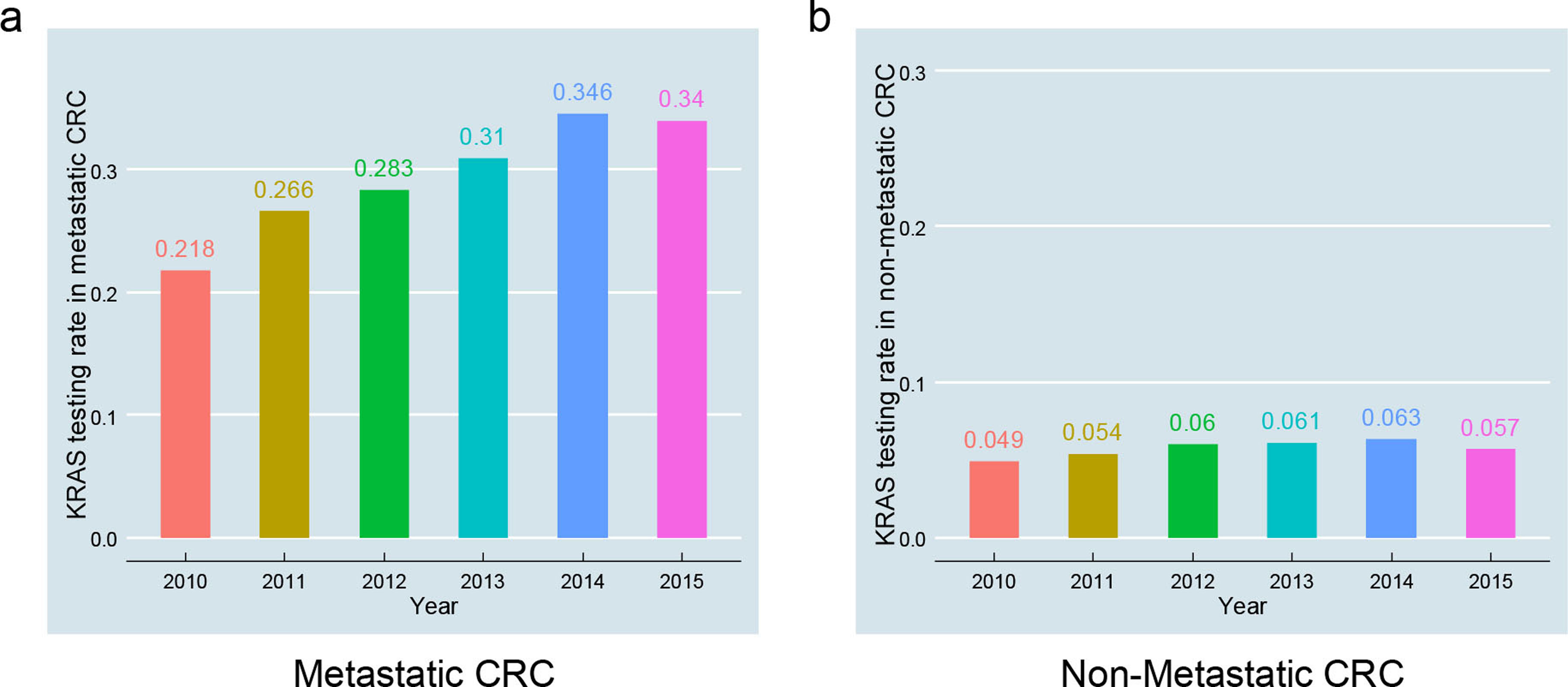

Supplement: Supplemental Information 2 [file peerj-08-9149-s002.png]
